# Cooperation loci are more pleiotropic than private loci in the bacterium *Pseudomonas aeruginosa*

**DOI:** 10.1073/pnas.2214827119

**Published:** 2022-10-03

**Authors:** Trey J. Scott

**Affiliations:** ^a^Department of Biology, Washington University in St. Louis, St. Louis, MO 63130

**Keywords:** pleiotropy, cooperation, kin selection, *Pseudomonas aeruginosa*

## Abstract

Pleiotropy may affect the maintenance of cooperation by limiting cheater mutants if such mutants lose other important traits. If pleiotropy limits cheaters, selection may favor cooperation loci that are more pleiotropic. However, the same should not be true for private loci with functions unrelated to cooperation. Pleiotropy in cooperative loci has mostly been studied with single loci and has not been measured on a wide scale or compared to a suitable set of control loci with private functions. I remedy this gap by comparing genomic measures of pleiotropy in previously identified cooperative and private loci in *Pseudomonas aeruginosa*. I found that cooperative loci in *P. aeruginosa* tended to be more pleiotropic than private loci according to the number of protein–protein interactions, the number of gene ontology terms, and gene expression specificity. These results show that pleiotropy may be a general way to limit cheating and that cooperation may shape pleiotropy in the genome.

Many loci are pleiotropic, where a pleiotropic locus is defined as one that affects multiple traits. Pleiotropy constrains evolution because mutations with beneficial effects on one trait can have deleterious effects on other traits. Several measures of pleiotropy have been used and shown to be associated with evolutionary constraint. Three examples are the number of protein interactions (1), the number of functional annotations (2), and how widely genes are expressed across tissues (3).

Pleiotropy is thought to limit cheaters and stabilize cooperation (4–7; see ref. 8 for a view on synergistic pleiotropy and ref. 9 for a dissenting view). Explaining how cheater evolution can be limited is a central question in the study of cooperation (10). Cheaters benefit from cooperation without paying the costs and are expected to outcompete cooperators. This cheater advantage can lead to the breakdown of cooperation unless cheaters are constrained (10).

Pleiotropy can limit cheaters when mutations at a locus underpinning a cooperative trait (cooperative locus) cause cheater phenotypes that come with harmful effects on other traits. One example of this comes from the social amoeba *Dictyostelium discoideum. D. discoideum* has a cooperative stage where 20% of cells sacrifice themselves to become stalk. The remaining cells become spores that are held up for dispersal by the stalk (10). This act of cooperation can be exploited by cheaters that contribute less to the stalk and increase their abundance in spores (10). Amoebae with *dimA* mutations are potential cheaters because they ignore the signal to become stalk (5). This should increase *dimA* mutant representation as spores. Instead, *dimA* mutants are excluded from spores when mixed with wild-type cells as a pleiotropic effect (5). This trade-off between becoming a stalk cell and entering spores limits the cheating ability of *dimA* mutants.

If pleiotropy at a cooperative locus limits cheaters and stabilizes cooperation, selection on cooperative groups may favor higher pleiotropy at cooperative loci relative to private (noncooperative) loci (4, 6). It is unknown whether cooperative loci are generally more pleiotropic than private loci, though this pattern has been observed in silico (4, 6). Prior studies of pleiotropy and cooperation in living organisms mostly focused on individual loci (5) or on gene coregulation (7). Such studies do not explicitly quantify pleiotropy or compare between cooperative and private loci. Private loci are an important control because they reflect the background pleiotropy and selection does not favor higher pleiotropy in these loci (4, 6).

Here, I take a genomic approach to compare loci involved in cooperative or private traits as categorized by Belcher et al. (11) in four *P. aeruginosa* gene sets. To identify cooperation loci, Belcher et al. (11) combined functional annotations and experimental data to identify gene products that act as a public good and can be cheated (often those that are secreted). These sets of loci have been constructed so that cooperative and private loci in a set are expressed under similar conditions and are similarly exposed to selection, all else being equal within a set (11). Belcher et al. (11) found evidence for relaxed selection in cooperation loci relative to private loci, a pattern that is consistent with kin selection. This is additional support that the cooperation and private categories capture something about the social effect of these loci. These sets of cooperative and private loci are therefore ideal for testing whether cooperative loci are more pleiotropic.

## Methods and Results

**Fig. 1.**
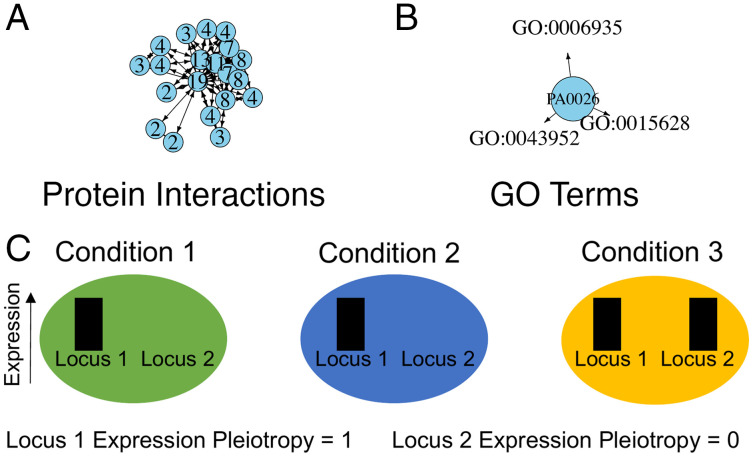
Examples of pleiotropy measures used in this study. (*A*) Number of protein interactions (STRING database). Arrows show predicted interactions that are summed to measure pleiotropy (the numbers on the nodes). (*B*) Number of biological process GO terms from the *P. aeruginosa* genome database. Arrows show annotations for an example locus with three GO terms. (*C*) Gene expression pleiotropy is calculated from gene expression data across multiple conditions. Locus 1 is expressed in every condition and is maximally pleiotropic. Locus 2 is specialized for a single condition and is minimally pleiotropic.

To test whether pleiotropy is higher in cooperation loci compared to private loci, I first used 315 quorum sensing (QS) loci (12) that were previously categorized as cooperative (*n* = 41) or private (*n* = 274) (Dataset S1) based on gene annotations and experimental data (11). As measures of pleiotropy, I used the number of protein interactions contained in the STRING database (13), the number of biological process Gene Ontology (GO) terms (14), and gene expression pleiotropy for each locus with available data (Fig. 1 and Dataset S2). More detailed methods can be found in *SI Appendix*, *Extended Methods*. These pleiotropy measures were not highly correlated and thus represent independent measures of pleiotropy (*SI Appendix*, *Extended Methods*).

**Fig. 2.**
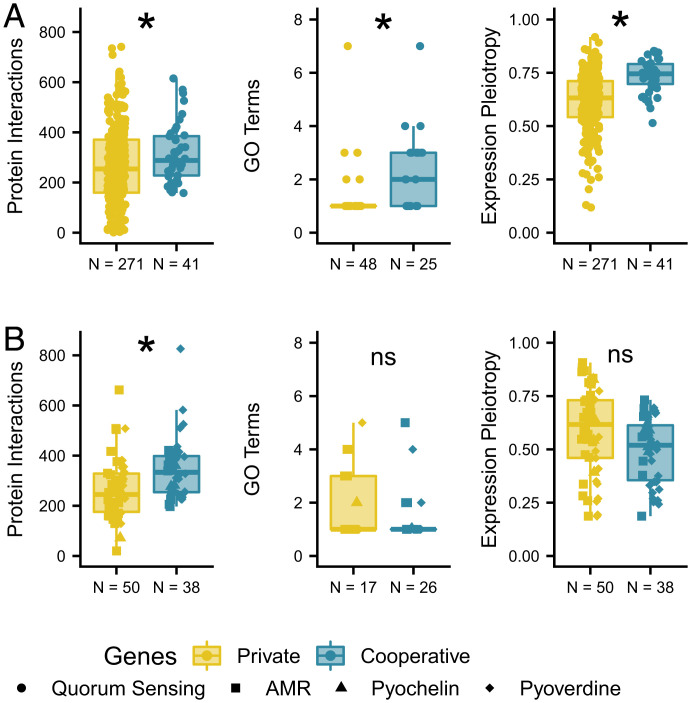
Cooperative loci tend to be more pleiotropic than private loci in the (*A*) quorum sensing and (*B*) additional sets of loci in *P. aeruginosa* (AMR = antibiotic resistance). Panels show the number of protein–protein interactions in the STRING database (*Left*), the number of GO terms (*Middle*), and the gene expression pleiotropy (*Right*). Number of loci with measures of pleiotropy are shown on the *x* axis. Colors are the same as in ref. 11 for easy comparison. *P ≤ 0.05.

I found that cooperative loci in the QS pathway were more pleiotropic across all three measures of pleiotropy (Fig. 2*A*). Cooperative loci had about 65 more STRING interactions on average than private loci (generalized linear model [GLM]; *P* = 0.016). Cooperative loci had two GO terms while private loci tended to have only one (GLM; *P* = 0.009). Gene expression pleiotropy was around 18% higher in cooperative loci than private loci (beta regression; *P* < 0.001).

To test whether these results apply beyond the QS pathway, I tested additional sets of cooperative and private loci (Dataset S3) in *P. aeruginosa* that were included in Belcher et al. (11). These sets consisted of antibiotic resistance genes (AMR) and pyochelin and pyoverdine genes that are involved in binding iron. To increase statistical power, I included set as a covariate (Fig. 2*B*; see *SI Appendix*, *Extended Methods*). Cooperative loci tended to have more STRING protein interactions than private loci (GLM; *P* = 0.008). However, cooperative and private loci were not different in terms of GO terms (GLM; *P* = 0.578) and expression pleiotropy (beta regression; *P* = 0.301).

## Discussion

Cooperation can break down because of the evolution of cheaters that benefit from cooperation without helping (10). The advantage of a cheater mutant at a cooperative locus can be limited if the cheater has pleiotropic effects on other important traits (4–7). Selection for cooperative groups may result in high pleiotropy at cooperative loci to limit cheaters (4, 6). Prior studies have focused on single loci (5), coregulation (7), and in silico analysis (4, 6) instead of measuring pleiotropy across many loci.

Using three independent measures of pleiotropy in *P. aeruginosa*, I found that pleiotropy tended to be higher in cooperative loci than in private loci regulated by QS (Fig. 2*A*). Only one comparison of the additional sets (Fig. 2*B*) resulted in more pleiotropy in cooperative loci. Pleiotropy may thus be higher only in QS loci or the effect of pleiotropy in the additional sets is detectable only as measured by STRING interactions. The additional sets also consist of fewer loci, which could have limited my ability to detect an effect.

My finding of increased pleiotropy in cooperation loci (Fig. 2) may strengthen the conclusions of Belcher et al. (11). This study found relaxed selection in cooperation loci relative to private loci consistent with predictions from kin selection theory. Belcher et al. (11) assumed that expression would be similar between cooperation and private loci because they are part of the same pathway. This would rule out relaxed selection due to cooperation loci being conditionally expressed in only some conditions (15). My expression pleiotropy measure shows that conditional expression does not explain the signals of relaxed selection in cooperative QS loci, since these were expressed across a wider set of conditions (Fig. 2*A*). Pleiotropy also affects patterns of selection, but it should be in the opposite direction of kin selection. Pleiotropy is associated with signals of conservation (2) and stabilizing selection (16), which should decrease genetic diversity and divergence. My results thus mean that the signal of kin selection in ref. 11 may be an underestimate, since increased pleiotropy in cooperation loci should dampen the signal of relaxed selection.

Theoretical studies have disagreed about the direction of causality between cooperation and pleiotropy and whether pleiotropy is able to stabilize cooperation if pleiotropy itself can evolve (4, 6, 8, 9). An interesting possibility that deserves more study is that cooperation creates the conditions for increased pleiotropy (6, 9), possibly through the evolution of more complex regulatory architectures underlying cooperative traits. While the data in this study cannot resolve these theoretical questions, they show that pleiotropy and cooperation are linked in *P. aeruginosa*. Cooperation may thus shape patterns of pleiotropy in the genomes of other cooperative organisms through its link with high pleiotropy.

## Supplementary Material

Supplementary File

Supplementary File

Supplementary File

Supplementary File

## Data Availability

Data for this analysis is included in the supporting information. Data and R code for analysis can also be found at www.gitlab.com/treyjscott/kin_selection_pleiotropy (17). All other study data are included in the article and/or supporting information.
