# Supplementary material for: Cooperation loci are more pleiotropic than private loci in the bacterium Pseudomonas aeruginosa
Source: Proc Natl Acad Sci U S A. 2022 Oct 3;119(41):e2214827119. doi: 10.1073/pnas.2214827119 (PMC9564939; doi:10.1073/pnas.2214827119)
Supplement: Supplementary File [file pnas.2214827119.sapp.pdf]

PNAS

Supplementary Information for

Cooperation loci are more pleiotropic than private loci in the bacterium *Pseudomonas aeruginosa*

Trey J. Scott
Department of Biology, Washington University in St. Louis

Corresponding author: Trey J. Scott
Email: tjscott@wustl.edu
Orcid ID: 0000-0001-6609-9638

This PDF file includes:

SI Extended Methods
SI References

Other supplementary materials for this manuscript include the following:

Datasets S1 to S3

SI Extended Methods

Gene Sets

I gathered 315 quorum sensing genes from Schuster et al. (1) and categorized 41 of these genes as cooperative from Belcher et al. (2). The remaining genes were classified as private genes that did not have a social function (Dataset S1). Additional pyochelin, pyoverdine, and AMR cooperative and private genes were also gathered from Belcher et al. (2) and are provided in Dataset S2.

Pleiotropy

I investigated three measures of pleiotropy (Dataset S3). First, I used predicted protein-protein interactions for *P. aeruginosa* PAO1 downloaded from the STRING database version 11.5 on May 6th, 2022 (3). STRING contains predicted protein interactions collected from high-throughput experiments, text mining, and other resources. These interactions may not necessarily involve physical interactions between proteins but should convey functional relationships. STRING entries have confidence scores that provide a measure of quality for interaction predictions. I incorporated this measure in statistical models by weighting according to the average score for a protein's combined interactions.

My second measure of pleiotropy was the number of non-redundant biological process gene ontology annotations for *P. aeruginosa* PAO1. These annotations convey information about the functions that a gene has or is predicted to have (4). These data were downloaded on April 4th 2022 from the *P. aeruginosa* genome database version 20.2 (5), which updates annotations based on new results published on *P. aeruginosa*.

My final measure was gene expression pleiotropy, a measure of how widely genes are expressed across conditions. This measure is useful in addition to the above measures from databases because it should be free from any biases associated with how loci are annotated. I calculated gene expression pleiotropy as 1 - τ, where τ is a common measure of gene expression specificity (6). τ ranges from 0, when a gene is expressed in all conditions tested, to 1, where the gene is expressed in only 1 condition and is calculated for each gene as

$$\tau = \frac{\sum_i \left(\left(1 - ln(x_i)\right) / ln(x_{max}) \right)}{N-1},$$

where N is the number of conditions, x_i is the expression level in conditions *i*, and x_{max} is the maximum expression across all conditions (7). τ is usually calculated across different kinds of tissues. Since *P. aeruginosa* does not have conventional tissues, I instead used gene expression data (GSE55197) from strain PA14 grown in 14 different conditions (8). I normalized raw transcripts using DESeq2 (9). To ensure that log expression was positive, I manually changed the minimum expression to 1.

Statistics

To determine whether the three pleiotropy measures were independent, I checked for correlations using Spearman's ρ. Correlations were weak ranging from -0.015 between GO terms and expression pleiotropy to -0.346 between protein interactions and expression pleiotropy. The correlation between GO terms and protein interactions was 0.039. These pleiotropy measures were thus relatively independent.

To test whether cooperative genes were more pleiotropic than private genes for the quorum sensing pathway, I used generalized linear models (GLMs). For protein interactions and GO terms, I fit models with Poisson errors. If I detected overdispersion, I fit quasi-Poisson and

negative binomial models for the final analysis. I conservatively reported the highest p-value between quasi-Poisson and negative binomial models if more than one model was fit. To include STRING confidence scores (see above), I weighted protein interaction models by the average score of its interactions. For gene expression pleiotropy, I used beta regression (10) to account for this measure being bounded from 0 to 1. To calculate means and p-values from statistical models, I used the *emmeans* package (11). I performed statistical tests in R (12) (version 4.1.2).

To test for differences between cooperative and private genes for the additional gene sets, I again used GLMs as above. I included the pathway (pyoverdine, pyochelin, or AMR) as a covariate in models, but compared means only between cooperative and private genes.

SI References:

1. M. Schuster, C. P. Lostroh, T. Ogi, E. P. Greenberg, Identification, Timing, and Signal Specificity of *Pseudomonas aeruginosa* Quorum-Controlled Genes: a Transcriptome Analysis. *J. Bacteriol.* **185**, 2066–2079 (2003).
2. L. J. Belcher, A. E. Dewar, M. Ghoul, S. A. West, Kin selection for cooperation in natural bacterial populations. *Proc. Natl. Acad. Sci.* **119**, e2119070119 (2022).
3. D. Szklarczyk, *et al.*, The STRING database in 2021: customizable protein–protein networks, and functional characterization of user-uploaded gene/measurement sets. *Nucleic Acids Res.* **49**, D605–D612 (2021).
4. Gene Ontology Consortium, The gene ontology resource: 20 years and still GOing strong. *Nucleic Acids Res.* **47**, D330–D338 (2019).
5. G. L. Winsor, *et al.*, Enhanced annotations and features for comparing thousands of Pseudomonas genomes in the Pseudomonas genome database. *Nucleic Acids Res.* **44**, D646–D653 (2016).
6. I. Yanai, *et al.*, Genome-wide midrange transcription profiles reveal expression level relationships in human tissue specification. *Bioinformatics* **21**, 650–659 (2005).
7. J. E. Mank, L. Hultin-Rosenberg, M. Zwahlen, H. Ellegren, Pleiotropic Constraint Hampers the Resolution of Sexual Antagonism in Vertebrate Gene Expression. *Am. Nat.* **171**, 35–43 (2008).

8. A. Dötsch, *et al.*, The Pseudomonas aeruginosa Transcriptional Landscape Is Shaped by Environmental Heterogeneity and Genetic Variation. *mBio* **6**, e00749-15 (2015).

9. M. I. Love, W. Huber, S. Anders, Moderated estimation of fold change and dispersion for RNA-seq data with DESeq2. *Genome Biol.* **15**, 1–21 (2014).

10. B. Grün, I. Kosmidis, A. Zeileis, Extended Beta Regression in *R* : Shaken, Stirred, Mixed, and Partitioned. *J. Stat. Softw.* **48** (2012).

11. R. Lenth, H. Singmann, J. Love, P. Buerkner, M. Herve, Package 'emmeans' (2019).

12. R Core Team, R: A language and environment for statistical computing (2013).
